# Astrocytic Alterations and Dysfunction in Down Syndrome: Focus on Neurogenesis, Synaptogenesis, and Neural Circuits Formation

**DOI:** 10.3390/cells13242037

**Published:** 2024-12-10

**Authors:** Beatrice Uguagliati, Mariagrazia Grilli

**Affiliations:** Laboratory of Neuroplasticity, Department of Pharmaceutical Sciences, University of Piemonte Orientale, 28100 Novara, Italy

**Keywords:** astrocyte, down syndrome, neurodevelopment, neurogenesis, astrogliogenesis, synapses

## Abstract

Down syndrome (DS) is characterized by severe neurodevelopmental alterations that ultimately lead to the typical hallmark of DS: intellectual disability. In the DS brain, since the prenatal life stages, the number of astrocytes is disproportional compared to the healthy brain. This increase is due to a shift from neuron to astrocyte differentiation during brain development. Astrocytes are involved in numerous functions during brain development, including balancing pro-neurogenic and pro-gliogenic stimuli, sustaining synapse formation, regulating excitatory/inhibitory signal equilibrium, and supporting the maintenance and integration of functional neural circuits. The enhanced number of astrocytes in the brain of DS individuals leads to detrimental consequences for brain development. This review summarizes the mechanisms underlying astrocytic dysfunction in DS, and particularly the dysregulation of key signaling pathways, which promote astrogliogenesis at the expense of neurogenesis. It further examines the implications of astrocytic alterations on dendritic branching, spinogenesis and synaptogenesis, and the impact of the abnormal astrocytic number in neural excitability and in the maintenance of the inhibitory/excitatory balance. Identifying deregulated pathways and the consequences of astrocytic alterations in early DS brain development may help in identifying new therapeutic targets, with the ultimate aim of ameliorating the cognitive disability that affects individuals with DS.

## 1. Overview of Down Syndrome

Down syndrome (DS) is the most common viable chromosomal abnormality occurring in humans. This genetic condition, characterized by the presence of all or part of a third copy of chromosome 21, occurs in approximately 0.45% of conception [1,2]. Even though DS presents a range of clinical features, intellectual disability is one of its most significant and debilitating characteristics [3]. 

Numerous studies have focused on the neuroanatomical correlates of the cognitive impairment associated with DS. Imaging studies of the fetal brain have described a reduced frontal lobe size; smaller cortical plates, subcortical parenchyma, and cerebellum, larger lateral and fourth ventricles; and reduced white matter [4,5,6]. The DS fetal cortex presents a delay in the emergence of lamination and has a disorganized appearance [7]. These morphological alterations appear to be caused by defects in brain cellularity; postmortem studies also describe severe neurogenesis impairments across multiple fetal brain regions, alongside a reduced number of radial glial cells (RGCs) and increased apoptosis [7,8,9,10,11,12]. Additionally, the fetal DS brain exhibits alterations in oligodendrocyte progenitors and astrocyte production during gestation [8,13,14,15]. Furthermore, an extensive postmortem characterization of the fetal DS brain shows reduced levels of several neurotransmitters: gamma-aminobutyric acid (GABA), the main neurotransmitter responsible for the excitatory neurotransmission in the developing brain; taurine, which acts as a neurotropic factor during brain development; dopamine, which is fundamental for the establishment of synaptic contacts; and serotonin (5-HT), which is crucial for proliferation, differentiation, migration, and synaptogenesis [16]. After birth, most of these prenatal alterations are retained. Imaging studies report that children with DS have a reduced brain volume, with smaller frontal, parietal, and temporal lobes; a smaller hippocampus, brainstem, and cerebellum; and greater parahippocampal gyrus volume, along with reduced cortical gyrification and white matter reduction [3,6,17]. Studies of brain cellularity showed a reduced number of neurons in the cortex, hippocampus, and cerebellum [18,19,20] accompanied by cortical and hippocampal increase in astrocytic markers [21]. Moreover, the dendritic sprouting typically observed during the early postnatal period is absent in children with DS, who, in contrast, are affected by a progressive reduction in dendritic arborization and dendritic spine number, which contributes to the impaired neuronal connectivity typical of DS [22,23,24,25]. 

Astrocytes are increasingly recognized as central players in several aspects of neurodevelopment. They contribute to the balance of the neuron-to-astrocyte ratio, support synapse formation and neurite outgrowth, and supervise the establishment of functional neural circuits. Astrocytic dysfunctions have been linked to various neurodevelopmental disorders, including DS. This review aims to provide an overview of the existing information regarding the major astrocytic alterations that affect the developing DS brain and their impact on the proper brain maturation, with particular attention paid to neurogenesis, synaptogenesis, and neural circuits formation. In particular, this work starts with a brief overview of the role of astrocytes in physiological brain development and their importance in neurogenesis, gliogenesis, synapse formation, and the establishment and maintenance of neural circuits. Thereafter, the review is dedicated to DS astrocytic alterations and their implication for brain development from prenatal stages. It highlights the key pathways contributing to the neuron-to-astrocyte imbalance in the trisomic brain, as well as the role of astrocyte in defective dendritogenesis and spinogenesis. Furthermore, it examines how astrocytic alterations impact the establishment of neural circuits, neuronal excitability, and the excitatory/inhibitory imbalance observed in the DS brain. 

The review process entailed a comprehensive search of PubMed, Scopus, and Google Scholar, using specific keywords, alone or combined, including “astrocyte”, “neurodevelopment”, “Down syndrome”, “neurogenesis”, “synapses”, “dendritic spine”, and “neurotransmitters”. Titles, abstracts, and full texts were reviewed according to the following inclusion criteria: thematic relevance, publication in peer-reviewed journals, and studies published in English. Exclusion criteria included findings from adult cellular models, adult populations, adult animal models. Thematically relevant findings were analyzed and synthesized to extract key findings summarized in this review.

## 2. Astrocyte in Physiological Brain Development

During prenatal development, astrocytes are derived from the differentiation of RGCs, which have the ability to self-renew and differentiate into both neurons and glial cells. The transition of RGCs from neurogenesis to astrogliogenesis occurs during the late gestation and early postnatal stages and is regulated by pro-gliogenic factors such as Janus kinase/signal transducers and activators of transcription (JAK/STAT) and Notch signaling pathways, as well as via direct cell-to-cell contact with other astrocytes [26,27,28,29,30,31]. Differently, in the postnatal brain, the majority of astrogliogenesis is driven by the symmetric division of mature astrocytes, and the newly formed astrocytes functionally integrate into the existing glial network [31]. Astrocytes play a crucial role in regulating the development of the nervous system and are involved in the maintenance of the delicate balance between pro-neurogenic and pro-gliogenic stimuli [32]. By releasing factors such as wingless-type MMTV integration site family-3 (WNT), insulin-like growth factor binding protein (IGFBP), and interleukins (IL), they regulate neuronal differentiation of progenitor cells [33,34]. In addition to their role in neurogenesis regulation, astrocytes are also essential factors in synapse formation and modulation. Astrocyte-secreted synaptogenic factors, such as thrombospondin-1 (TSP-1), promote the formation of excitatory synapses by binding to the α2δ-1 receptor on neurons [35]; the expression of pentraxin-3 (PTX3) and glypican-4 (GPC-4) and -6 by astrocytes during early postnatal development enhances excitatory synapse formation [36,37]. Conversely, astrocyte-released transforming growth factors-β1 (TGF-β1) regulates inhibitory synapse formation through the calcium/calmodulin-dependent protein kinase type II (CaMKII) pathway [38]. Astrocytic neuronal cell adhesion molecule (NrCAM), by the binding to NrCAM–gephyrin complexes on postsynaptic neurons, also contributes to the formation and function of inhibitory synapses [39]. Moreover, astrocytic expression of ephrin-B1 (EFNA-B1) regulates the excitatory/inhibitory (E/I) balance in the developing hippocampus, reducing excitatory synapse formation and enhancing synaptic inhibition [40]. Through their influence on neurons and synapses, astrocytes are crucial for the maintenance and integration of functional neural circuits. Unlike neurons, astrocytes do not generate action potentials. Indeed, they exhibit intracellular calcium events in response to neuronal activity, which, in turn, trigger the release of neuroactive gliotransmitters. The astrocytic release of molecules such as adenosine, glutamate, and GABA, along with the modulation of synaptic activity via metabotropic and ionotropic receptors, significantly contributes to brain plasticity, memory retention, and long-term potentiation (LTP) [41,42,43,44,45,46].

## 3. Astrocyte Alterations in Down Syndrome

As briefly reported in the introduction of this work, individuals with DS exhibit an increased number of astrocytes, which also tend to be larger and show elevated expression of astroglial markers such as S-100β and glial fibrillary acidic protein (GFAP) [21,47]. Postmortem studies showed that these astrocytic alterations are evident from the prenatal stages of development. An increased number of astrocytes and RGCs have been reported in the frontal lobe of DS fetuses at 18–20 gestational weeks (GW) [14]. During GW 17–21, DS fetuses also display a higher percentage of GFAP-positive cells at the expenses of neuronal cells in the fusiform gyrus, inferior temporal gyrus, and subiculum [7,8]. On the contrary, a lower expression of GFAP-positive cells has been reported in the hippocampus and temporal lobe of DS fetuses during GW 18–26 [13]. These discrepancies in the neuron-to-astrocyte ratio suggest a shift from neurogenesis to gliogenesis. This tendency toward astroglial formation is further supported by a study on induced pluripotent stem cells (iPSCs) derived from monozygotic twins discordant for Trisomy 21: DS-iPSC-derived cells show increased expression of astrocytic markers, such as GFAP, S-100β, and vimentin, and a reduced expression of neuronal markers [48]. It is important to note that the astrocytic alterations displayed in the prenatal period are retained after birth. Indeed, a postmortem study in children with DS showed a reduction in the number of astroglial interlaminar processes, accompanied by focal astrogliotic changes that progress with age [49]. The described imbalance of the astrocyte/neuron number that affects the brain of individuals with DS since prenatal development and continues throughout the individual’s whole life may be one of the primary contributors to the brain developmental alterations that result in the intellectual disability characteristic of DS.

### 3.1. Effects on Neurogenesis

**Figure 1:**
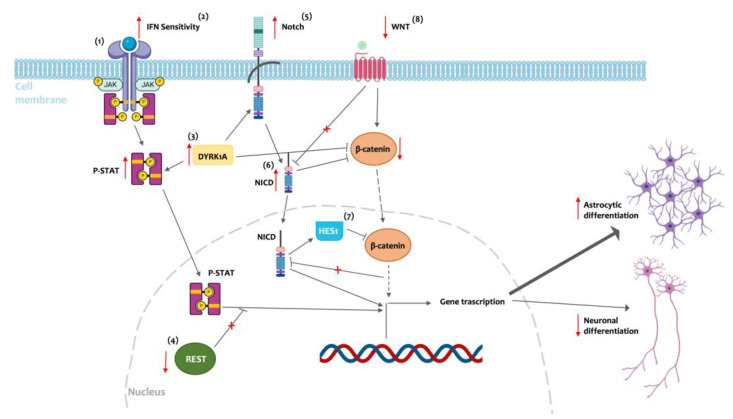
Schematic representation illustrating the neurons-to-astrocytes shift typical of the developing brain in Down syndrome (DS). (1) Hyperactivation of the Janus kinase/signal transducers and activators of transcription (JAK/STAT) pathway promotes astrocytic differentiation, a process influenced by multiple factors, including (2) hypersensitivity to interferon (IFN), (3) overexpression of dual specificity tyrosine-phosphorylation-regulated kinase-1A (DYRK1A), and (4) downregulation of repressor element-1 silencing transcription factor (REST). Additionally, upregulation of the Notch protein in DS (5), partially mediated by DYRK1A overexpression (3), increases the levels of Notch intracellular domain (NICD) (6), which translocates into the nucleus, further promoting astrocytic fate during differentiation. Moreover, NICD promotes the expression of HES1 (7), fostering gliogenesis through the suppression of pro-neurogenic signals. The reduction of the wingless-type MMTV integration site family (WNT)/β-catenin pathway observed in the DS brain (8) participates in the neuron-to-astrocyte shift. Furthermore, DS overexpression of DIRK1A (3) and HES1 (7) contribute to the reduction of the β-catenin pro-neurogenic pathway, which, in turn, promotes NICD signal further fostering astrogliogenesis. Plain arrows indicate the signaling direction, dotted arrows indicate the signaling reduction, and red arrows indicate the typical DS alterations. Created with Biorender.com.

The described enhanced astrocytic number that affects the brain of individuals with DS runs in parallel with a reduction of the neuronal population. The decrease in neuron number is attributable to many causes, among which is a reduced neuronal differentiation in favor of astrocytes [50,51]; several mechanisms have been proposed as the cause of neural progenitor fate with respect to the astroglia phenotype (Figure 1).

The JAK-STAT pathway is one of the principal candidates for astrogliogenic differentiation. In samples from DS children, activation of JAK autophosphorylation leads to the signaling cascade through STATs, which, in turn, promotes the activation of astrocytic genes, among which are GFAP and S-100β [52]. Studies of the JAK-STAT pathway in the Ts1Cje mouse model (a widely used mouse model of DS, trisomic for 94 genes located on mouse cromosome 16 [53]), which is characterized among other typical DS neurodevelompental alterations by an increased astrocyte number compared to wild-type animal, showed an alteration of JAK and STAT phosphorylation during prenatal and early postnatal development in the brain and in cortex-derived neurospheres [54,55]. The JAK-STAT signaling pathway is activated by several ligands, among which are ILs and interferons (IFNs). Interesting, four IFN and IL receptors genes, namely, IFNα-receptor 1 and 2 (IFNAR-1, IFNAR-2), IFNγ-R2 (IFNGR2), and IL-10 receptor β (IL10RB), are located on human chromosome 21 (HSA21), and in individuals with DS, their expression is ~1.5 times higher (proportional to the gene dosage effect of the trisomy), leading to an increased IFN sensitivity and an enhanced JAK-STAT pathway activation [52]. Another key regulator of the JAK-STAT pathway with critical importance in the context of DS is the dual-specificity tyrosine-phosphorylation-regulated kinase 1A (DYRK1A). The DYRK1A gene is also located on HSA21 and overexpressed in DS. The overexpression of DYRK1A in mouse NPCs promotes differentiation towards astrocytic fate, while DYRK1A depletion in Ts1Cje-derived NPCs reduces the number of NPCs differentiating into astrocytes. Furthermore, in Ts1Cje mice, depletion of DYRK1A attenuated the augmented astrocytic production during corticogenesis [54]. Notably, in mouse neocortex, the number of neurons is inversely correlated with the DYRK1A copy number, enforcing the putative role of DYRK1A in the alteration of the neuron-to-astrocyte number during brain development [56]. Additionally, the repressor element-1 silencing transcription factor (REST) is involved in the control of gene expression in neuronal cells, and among REST-targets is the JAK-STAT signaling pathway. In neurospheres derived from DS fetuses, REST protein levels are lower, and this can lead to a further augmented JAK/STAT signal [57]. Another candidate for the neuron-to-astrocyte switch that characterizes the brain of individuals with DS is the Notch protein. The cleavage of Notch by secretase releases the Notch intracellular domain (NICD), which translocates into the nucleus, activating effector proteins that foster astroglial differentiation. Postmortem studies reveal that the Notch gene is upregulated in the fetal DS brain [58]. In the Ts1Cje mice, Notch is downregulated during embryonic stages, while after birth, postnatal day 1 (P1), the expression of Notch is upregulated in the hippocampus, and at P30, it is downregulated in the hippocampus but upregulated in the neocortex, supporting astrocytic differentiation following maturation trajectories [59]. Taken together, the upregulation of JAK-STAT and Notch signaling may synergistically contribute to astrogliogenesis, thereby suppressing neurogenesis and oligodendrogenesis in the DS brain. Another pathway that might be involved in the disproportional astrogliogenesis that characterizes DS is the WNT/β-catenin pathway. WNT signaling directs cell proliferation and cell fate determination during embryonic development [60]. In iPSCs generated from a young individual with DS, the activation of WNT/β-catenin pathway is reduced [61], and it has been demonstrated that loss of β-catenin in mouse-derived neural progenitors causes a shift toward astroglial differentiation [62], thus corroborating the hypothesis of WNT/β-catenin pathway involvement in the neuron-to-astrocyte switch in DS. To recapitulate, the number of neurons and astrocytes is finely regulated in the healthy brain. The alteration of gene dosage and the dysregulation of signaling pathways due to trisomy 21 contribute to the shift from neurogenesis to astrogenesis, leading to a cascade of subsequent brain alterations that contribute to intellectual disability in DS.

### 3.2. Effects on Synaptogenesis

**Figure 2:**
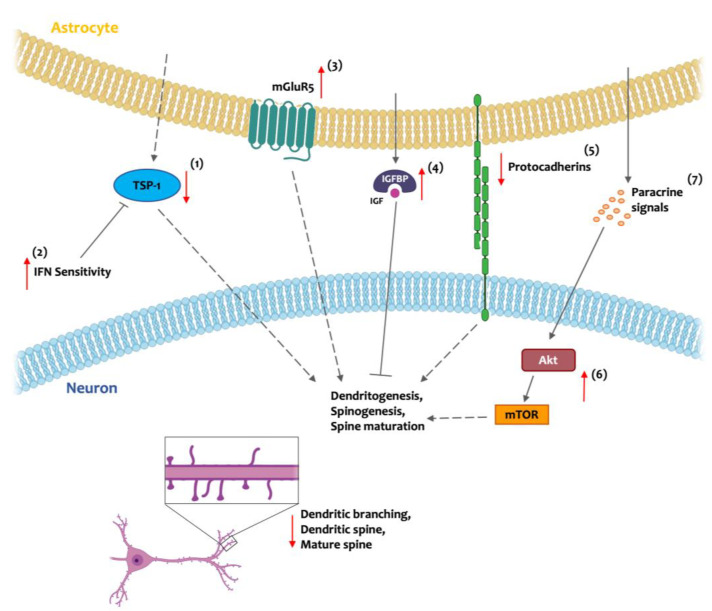
Schematic representation of the effects of astrocytic alterations on synaptogenesis in Down syndrome (DS) brain development. Reduced dendritic branching and impaired dendritic spine maturation in DS are associated with several astrocytic factors. DS astrocytes produce lower levels of the synaptogenic factor thrombospondin-1 (TSP-1) (1), partially attributable to increased interferon (IFN) hypersensitivity (2). In addition, elevated astrocytic expression of metabotropic glutamate receptor-5 (mGluR5) (3), which is involved in astrocyte–synapse signaling, further contributes to synaptogenesis abnormalities; similar effects are caused by increased astrocytic secretion of insulin-like growth factor binding protein (IGFBP2) (4), which interacts with insulin-like growth factor (IGF) and interferes with synaptogenesis regulation. Moreover, reduced expression of adhesion molecules, such as astrocytic protocadherins (5), impairs cell adhesion/recognition processes during synapse formation. Finally, DS neurons exhibit higher Akt/mammalian target of rapamycin (Akt/mTOR) signaling (6), a pathway involved in synapses regulation, partially mediated by astrocytic secreted paracrine signals (7). Plain arrows indicate the signaling direction, dotted arrows indicate signaling reduction, and red arrows indicate the typical DS alterations. Created with Biorender.com.

Post mortem studies have shown that starting from early life stages, individuals with DS experience a progressive reduction in dendritic arborization and dendritic spine density, accompanied by impaired dendritic spine maturation [23,24,25,63]. Astrocytes, through secreted factors, as well by direct cell contact, are important regulators of synaptic formation and dendritic arborization [64] (Figure 2). The astrocytic-derived synaptogenic factor TSP-1 is a modulator of dendrites and dendritic spine development. In the conditioned media of human DS astrocytes, TSP-1 levels are markedly reduced, and reduced levels of TSP-1 are responsible for dendritic branches with lower complexity and fewer and immature dendritic spines in a human–rat coculture system; such conditions can be reverted through TSP-1 administration [65,66,67]. One of the possible causes behind TSP-1 deficiency in DS can be attributable to DS hypersensitivity to IFN; IFN receptor genes are located on HSA21 [52,65]. In addition, the Akt/mammalian target of the rapamycin (Akt/mTOR) signaling pathway, a ubiquitous serine/threonine kinase pathway involved in synaptogenesis, is upregulated in the brain tissue of young adults with DS [68] and in the hippocampal neurons of Ts1Cje mice [69]. This hyperactivation may be partially mediated by astrocytes through paracrine signaling and contributes to the disruption of synapses formation [70]. Moreover, astrocytes derived from Ts65Dn mice (one of the most extensively used and well-characterized mouse models of DS, carrying a trisomy of mouse chromosome 16, including orthologs of approximately half of the genes on HSA21 [71]) display elevated levels of IGFBP-2, which, through its interaction with insulin-like growth factor (IGF), inhibits neurite outgrowth [34]. IGFBP-2 is required during brain development for proper neural circuit formation and LTP [72]; thus, dysregulation of IGFBP-2 levels in the trisomic brain may aggravate the altered synaptogenesis and contribute to alterations in neuronal connectivity. Furthermore, RNA-sequencing analysis of astrocytes derived from DS patients reveals dysregulation of cell adhesion and extracellular matrix molecules, with notable alterations in the expression of several protocadherins (PCDHs) [73], confirming previous findings regarding γ-PCDH downregulated in postmortem DS fetal cortex [74]. PCDHs take part in adhesion/recognition processes during synaptogenesis [75]; altered levels of these proteins may therefore represent another astrocytic-derived contributor to the altered dendritic arborization and synaptogenesis that affect the brain of individuals with DS. Additionally, a postmortem study in the fetal DS brain showed an astrocytic increased expression of metabotropic glutamate receptor 5 (mGluR5), which persisted postnatally [76]. mGluR5 is involved in astrocyte/synapse cross talk, and an altered mGluR5 expression can contribute to the altered synaptogenesis in DS.

### 3.3. Effects on Neural Circuits Development

**Figure 3:**
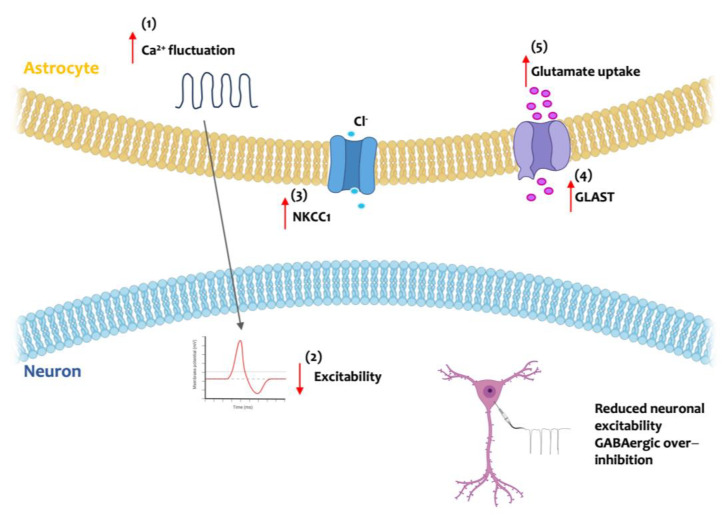
Schematic representation of the effects of astrocytic alterations on neuronal connectivity and neuronal circuits in Down syndrome (DS) brain development. DS exhibit increased calcium fluctuations (1), which can reduce neuronal excitability (2). Moreover, astrocytes play a role in the excitatory/inhibitory (E/I) imbalance typical of DS; overexpression of sodium potassium chloride cotransporter1 (NKCC1) (3) fosters DS GABAergic over-inhibition, further amplified by increased expression of glutamate–aspartate transporter (GLAST) (4) on astrocytic membranes, which, in turn, increases glutamate uptake (5). Plain arrows indicate the signaling direction; red arrows indicate the typical DS alterations. Created with Biorender.com.

The aforementioned alterations in dendrite structure, dendritic spine formation, and synaptogenesis that occur during neurodevelopment in individuals with DS significantly impact the establishment of neural circuits. The abnormal astrocytic population that affects the DS brain plays a detrimental role, further exacerbating alterations in neuronal connectivity and network functionality (Figure 3). Astrocytic calcium fluctuations are essential for the physiological regulation of neuronal excitability and synaptic transmission. Astrocytes derived from DS patients iPSCs exhibit more frequent spontaneous calcium fluctuations compared to isogenic astroglia. Enhanced calcium activity in DS astrocytes reduces the excitability of co-cultured neurons; conversely, suppression of the astrocytic spontaneous calcium activity rescues neuronal excitability [77]. In contrast, another study reported no calcium activity alterations in human derived-DS astrocyte [73]; likely, additional studies are needed to clarify the effects of DS on astrocytic calcium dynamics. Indeed, these studies were performed in different cell lines. This, in addition to the fact that neonatal Ts65Dn mice exhibit elevated cortical astrocyte calcium activity similar to that seen in embryonic astrocytes from Ts16 mice [78] (one of the first mouse models to mimic some of the genetic and phenotypic characteristics of DS; it carries a duplication of a portion of mouse chromosome 16, which is similar to part of HSA21 [79]), suggests the need for of further investigation. A well-documented characteristic of the DS brain is the imbalance of E/I signals that has been described since early development and has detrimental effects on neurodevelopment. Postmortem studies revealed GABA reductions during prenatal stages [16], while the postnatal brain, both in DS individuals and in DS mouse models, is affected by GABAergic over-inhibition, attributable to an increased number of GABAergic neurons and synapses, as well as to increased chloride gradients [80]. The mechanism underlying DS GABA alterations during brain development is still not well understood. However, since the sodium potassium chloride cotransporter-1 (NKCC1) is one of the channels responsible for controlling the inhibitory homeostasis of GABAergic signals during brain development [81], and NKCC1 is expressed in various cell types, including astrocytes [82], the enhanced number of astrocytes in the trisomic brain can contribute to augmented NKCC1 expression in DS and to GABAergic dysregulation. The other key player of the typical imbalance of E/I signals is glutamate. Glutamate levels are reduced in the DS brain with human trisomic astrocytes showing increased expression of the glutamate–aspartate transporter (GLAST), leading to higher glutamate uptake [83].

## 4. Conclusions

This review contributes to the existing literature by providing a comprehensive overview focused on the mechanisms underlying the dysregulation of the neuron-to-astrocyte ratio in DS and the consequences of an increased number of astrocytes, alongside astrocytic alterations caused by trisomy 21, in the developing brain. Astrocytes are increasingly recognized as central participants in neurodevelopment. They take part in numerous processes that lead to the building of the mature brain, such as in neurogenesis, in synapse formation, in the support and integration of functional neural circuits, and in the maintenance of central nervous system homeostasis. An abnormal number of astrocytes is detected in the brain of individuals with DS since the prenatal period, contributing to the neurodevelopmental alterations that ultimately lead to the intellectual disability associated with DS. Interestingly, other common comorbidities in DS may also be linked to astrocytic dysfunctions. The increased risk of epileptic seizures in individual with DS can be reconducted to neurotransmitter imbalance and GABAergic over-inhibition, in which astrocytes may take part [84]. Furthermore, astrocytic alterations in the motor cortex may impair the formation and maintenance of synapses crucial for motor learning and coordination, whose impairment is observed in DS [85]. Additionally, while astrocytic abnormalities during early developmental stages cannot be directly linked to the formation of amyloid plaques, early astrocytic dysfunctions may indirectly contribute to the later development of Alzheimer’s disease. Impairment of astrocytic function may indeed reduce the ability to clear amyloid-beta, thereby contributing to Alzheimer’s pathology [86]. The number of studies investigating the role of astrocytes in neurodevelopment and neurodevelopmental disorders is growing, reflecting increased awareness regarding the importance of non-neuronal players in early brain development, a window of particular susceptibility during which the foundations for the adult brain are built. However, the study of astrocytic alterations in DS still includes some aspects that remain unexplored. While the presence of a neuron-to-astrocyte imbalance is clear, research regarding the contribution of astrocytic malfunctioning to synaptogenesis, neuron excitability, and neurotransmission, as well as the mechanisms underlying astrocytic malfunction’s contribution to learning and memory impairment in DS, need to be deepened. Moreover, most therapeutical interventions under investigation for the treatment of brain developmental abnormalities in DS are focused on neurons. However, in view of the critical role of astrocyte in neurodevelopment, targeting specific aspects of astrocytic (dys)function could offer alternative avenues for therapeutic intervention. For instance, addressing altered pathways regulating astrocytic differentiation, such as JAK/STAT, Notch signalling, and WNT/β-catenin, could help in restoring the correct neuron–astrocyte balance in the DS brain, improving brain function. In addition, interventions targeting astrocytic calcium signalling and altered gliotransmission could potentially improve synaptogenesis, synaptic function, and circuits formation. To conclude, a more detailed understanding of the role of astrocytes in DS developmental alterations could pave the way for early interventions aimed at mitigating cognitive impairment and enhancing the quality of life of individuals with DS.
